# Day Care General Anaesthesia for a Child with Bardet-Biedl Syndrome

**DOI:** 10.1155/2010/239239

**Published:** 2010-08-01

**Authors:** Suresh Chittoodan, Suzanne Crowe

**Affiliations:** Department of Anaesthesia, Pain Management, and Intensive Care Medicine, Adelaide Meath and National Children's Hospital, Dublin 24, Ireland

## Abstract

Bardet-Biedl syndrome is a very rare autosomal-recessive disorder with pan-systemic effects. The perioperative period may be hazardous for patients with this disorder. We describe the presenting features and management of a child who was scheduled for elective ambulatory general anesthesia and discuss the relevant points for the busy anesthesiologist.

## 1. Introduction

Bardet-Biedl syndrome is an autosomal-recessive disorder characterized by cone-rod dystrophy, truncal obesity, postaxial polydactyly, cognitive impairment, male hypogonadotrophic hypogonadism, complex female genitourinary malformations, and renal dysfunction [1, 2]. It was formerly grouped with Laurence-Moon-Biedl syndrome but is now considered as a separate entity. Renal disease is a major cause of morbidity and mortality. The secondary features include speech disorder, developmental delay, ataxia, retinal abnormalities, cardiovascular abnormalities, diabetes mellitus, diabetes insipidus, and hepatic and dental involvements [1, 2]. There is a paucity of literature published on the anaesthetic management of patients with this syndrome.

## 2. Case Presentation

We present a report of 8-year-old girl with Bardet-Biedl syndrome who underwent MRI of her brain and spinal cord under day care general anaesthesia. Some of the challenging aspects of this case were the reduced ability to communicate or cooperate because of severe mental retardation and partial blindness, obesity with BMI 36 kg/m^2^, hypertension, poor renal function, and the limitations imposed by the unique MRI environment.

She was diagnosed to have Bardet-Biedl syndrome in early childhood. She had abnormal renal function due to the presence of a right-sided duplex kidney and a left-sided poorly functioning kidney. Her blood pressure was controlled with enalapril 0.15 mg/kg twice daily. She had postaxial polydactyly of both lower limbs and required assistance for all activities because of severe mental retardation and partial blindness. There was no history of cardiovascular anomalies or diabetes mellitus.

She was assessed in the day care unit and her mother reported that a previous attempt at sedation had made her daughter agitated and aggressive. She was premedicated with oral midazolam 0.5 mg/kg and ketamine 3 mg/kg. Twenty minutes later, standard MRI compatible monitors including pulse oximeter, electrocardiogram, and noninvasive blood pressure were applied. General anaesthesia was induced with O_2_, N_2_O, and sevoflurane. Mask ventilation was easy. Intravenous access was secured with a 22 G cannula on the dorsum of the right hand. At the time of intubation, we noticed a bifid epiglottis which is an extremely rare congenital anomaly. It has been reported in association with this syndrome [2]. The curved laryngoscope blade was changed to a straight blade, improving the view of the glottis and the trachea was then intubated with a cuffed 5.5 endotracheal tube and spontaneous ventilation was continued with sevoflurane in oxygen and nitrous oxide, fiO_2_ 0.5. On completion of the procedure, the trachea was extubated with the patient awake. There was no stridor in the recovery room, and she was discharged home on the same day. Throughout induction, maintenance, and emergence her blood pressure was well controlled with an average mean of 65 mmHg. In view of the incidental finding of bifid epiglottis, she was referred to a pediatric otolaryngologist for further evaluation.

## 3. Discussion

This syndrome is much commoner in the Middle East with an incidence of 1 : 13,500. In the rest of the world, the incidence is 1 : 160,000 with a male-to-female ratio of approximately 1.3 : 1. Children with this condition frequently require multiple anaesthetic procedures for both diagnostic and therapeutic measures including MRI, uro-gynaecological procedures, or corrective surgeries for limb deformities. Literature review revealed only two case reports about this rare syndrome, one in paediatric and the other in adult population.

Low and his colleague reported several anaesthetic problems associated with this syndrome including obesity with consequent problems with venous access and placement of local anaesthetic blocks [3]. We chose to intubate this patient's trachea because of her obesity. Sedative premedication with both midazolam and ketamine was utilised because of previous sedation failure in this child.

An adult case of Bardet-Biedl syndrome, dilated cardiomyopathy, and fractured right femur and tibia that successfully underwent open reduction and internal fixation under combined spinal and epidural anaesthesia was reported by Mahajan et al. [4]. Elbedour et al. reported 11 out of 22 patients (50%) had hypertrophy of the interventricular septum and dilated cardiomyopathy [5]. Because of this reported incidence, we would suggest it is prudent to consider a 12-lead ECG and echocardiogram during the preanaesthetic assessment.

In summary, general anaesthesia was safely performed on a day care basis in this patient with a rare congenital condition. Although no complication was encountered in our case, this syndrome has the potential for difficulties in managing the airway and the cardiovascular and renal systems.

##  Funding

There was no funding required for the preparation of this paper.

##  Confilct of Interest

The authors have no conflict of interest with the issues discussed in this letter.
